# Comprehensive Detection of Gas Plumes from Multibeam Water Column Images with Minimisation of Noise Interferences

**DOI:** 10.3390/s17122755

**Published:** 2017-11-29

**Authors:** Jianhu Zhao, Junxia Meng, Hongmei Zhang, Shiqi Wang

**Affiliations:** 1 Institute of Marine Science and Technology, Wuhan University, Wuhan 430079, China; jhzhao@whu.edu.cn (J.Z.); 2011301610337@whu.edu.cn (S.W.); 2 School of Geodesy and Geomatics, Wuhan University, Wuhan 430079, China; 3 School of Power and Mechanical Engineering, Wuhan University, Wuhan 430072, China; hmzhang@whu.edu.cn

**Keywords:** gas plumes, water column image, threshold detection, intersection operation, morphological constraint

## Abstract

Multibeam echosounder systems (MBES) can record backscatter strengths of gas plumes in the water column (WC) images that may be an indicator of possible occurrence of gas at certain depths. Manual or automatic detection is generally adopted in finding gas plumes, but frequently results in low efficiency and high false detection rates because of WC images that are polluted by noise. To improve the efficiency and reliability of the detection, a comprehensive detection method is proposed in this paper. In the proposed method, the characteristics of WC background noise are first analyzed and given. Then, the mean standard deviation threshold segmentations are respectively used for the denoising of time-angle and depth-angle images, an intersection operation is performed for the two segmented images to further weaken noise in the WC data, and the gas plumes in the WC data are detected from the intersection image by the morphological constraint. The proposed method was tested by conducting shallow-water and deepwater experiments. In these experiments, the detections were conducted automatically and higher correct detection rates than the traditional methods were achieved. The performance of the proposed method is analyzed and discussed.

## 1. Introduction

New generation multibeam echosounder systems (MBES), such as the Kongsberg EM710/EM302 [1,2,3] and Teledyne Reson 7125/8125 [4,5] can collect water column (WC) data. WC data include the full acoustic information from the MBES transducer to the seafloor [1] and provide an effective way to find underwater objects such as fish schools [4,6,7,8], wrecks [9,10,11], eelgrass [12], gas plumes [13,14], and internal ocean waves [15,16]. Gas plumes may be an indicator of the presence of gas hydrate. Methane hydrate is a clear energy source with potential importance as an energy source with environmental benefits [17]. The search for methane hydrate by detecting gas plumes from WC data is a new and low-cost alternative method to the traditional seismic exploration [2,18].

Gas plumes are formed by gas leakage [19,20,21]. Gas leakage may be modulated by temperature and pressure. The driving force of leakage is the production of methane at depth and resulting overpressure, possibly linked to fluid migration, and the buoyancy of the gas. Gas hydrate dissolution is another driver that is highly susceptible to temperature increases. Methane gas bubbles usually dissolve completely during their rise. Whether methane gas bubbles reach the sea surface depends on the water depth and initial bubble size. Many bubbles dissolve after rising a few dozens of meters [22]. The gas plumes can be found from the WC data and the rising process of gas plumes can be reflected in the consecutive MBES WC images [23,24].

Manual or automatic detection methods are often used for finding gas plumes from WC data. Generally, gas plumes have higher backscatter strengths (BSs) than does noise in the WC data and significant morphologic differences from noise in the WC images. Based on these characteristics, manual detection can find gas plumes in WC images. Manual detection is classic and convenient and is thus frequently adopted in the detections of gas plumes. However, manual detection is also variable because of subjective differences in experience and time consumption for finding gas plumes in large amounts of MBES WC data.

Many scholars have developed automatic detection algorithms to improve the detection efficiency. The threshold filtering approach is often adopted in the automatic detection of gas plumes. Veloso et al., [25] used simple threshold filtering, speckle noise removal, and manual editing in a 3D space to clean the WC data of a single-beam echosounder and segment targets from the WC images and quantified the bubble flow rates in deepwater. Marques et al., [26] proposed a layered threshold method for automatically determining the threshold. The pixels, whose BSs satisfy the threshold condition, can be found efficiently, but the pixels in the target boundary cannot be detected well, thereby leading to the inaccurate determination of the shape and size of the detected target. Urban et al., [27] studied the automatic detection of bubble streams from the MBES WC images by the median signal-based mask with a threshold and their spatiotemporal behavior. These researchers mentioned that the proposed median stacking method is only efficient when the artifacts in the WC image do not change in many consecutive swaths and would fail in the area where assuming a homogeneous flat seafloor is invalid. The above threshold filtering methods have shortcomings in determining the threshold automatically and accurately, thereby resulting in frequent detection interruption and low correct detection rates. Two classic threshold segmentation methods, namely, 2D Otsu and iterative methods [28], are frequently used for automatically determining the threshold and segmenting the targets from a side scan sonar image [29,30,31,32]. However, the methods may demonstrate low efficiency because of adopting the traditional exhaustive search method to calculate the threshold vector and low segmentation accuracy because of the detected sonar image polluted by the complicated WC noise [33]. The automatic detection methods [34,35] improve the efficiency compared with manual detection, but frequently demonstrate low correct detection rates when the WC image is polluted by the noise from the WC environment and interferences caused by the WC imaging mechanism.

A comprehensive detection method is proposed in this paper to improve efficiency and accuracy of gas plume detection. First, the characteristics of WC background noise are analyzed and provided. Then, the proposed method is described, including the threshold segmentation to the time- and depth-angle images, intersection operation to the two segmented images, and gas plume detection from the intersection image by the extracted morphological characteristics. The proposed method is tested by experiments and its performance is discussed. Several conclusions are drawn based on the experiments and discussions.

## 2. WC Image Characteristics 

### 2.1. WC Image

In the WC measurements of MBES such as EM710 and, EM122, the transducer transmits pulses with various frequencies in the different sectors, and receives the backscatter strength sequence of each beam in these sectors (the BS value represents the sample amplitude in dB). WC data are recorded into a binary file with the suffix *.wcd by MBES data acquisition software. To display these WC data as images, the *.wcd files must be decoded by referring to the Kongsberg EM series MBES datagram format [36]. Then, the data preprocessing, including the correction of sound velocity, the correction of transmission loss, and the positional calculation, is conducted for decoded WC data to obtain BS and its position of each echo. Via converting the BS to gray level, the WC image of a ping is formed by combining BSs of all sectors in the ping. The above process can be conducted by commercial software for MBES data processing such as CARIS HIPS or self-developed software.

**Figure 1:**
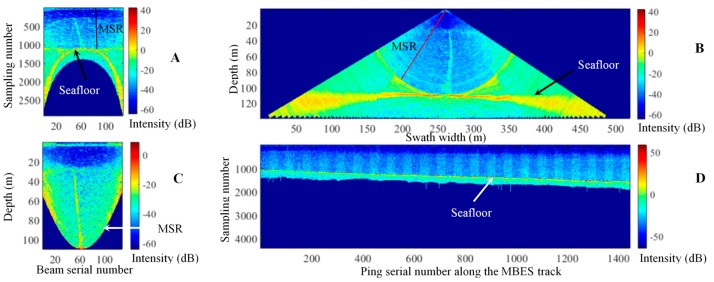
Different display modes of a WC image. (**A**–**D**) denote the T-A, D-T, D-A, and along-track images. The first three images are called across-track images.

Typically, WC data are shown as the along- or across-track images. Figure 1D depicts the along-track image of a beam; the along-track image is used to rapidly find targets when the beam goes through the targets. Furthermore, accurately obtaining the shapes and sizes of targets by using the along-track image is infeasible because of their only being scanned by a beam [1].

Time-angle (T-A) and depth-across track (D-T) images are two classic across-track images [1] and are often used for detecting targets and determining the shapes of the targets. In a T-A image (Figure 1A), the abscissa and the ordinate denote beam serial and sampling point numbers. In a D-T image (Figure 1B), the two axes indicate swath width and vertical distance from the transducer to its nadir. The T-A and D-T images permit that the location and shape of a target can be determined accurately by the D-T image but not the T-A image. A T-A image has smaller size than the corresponding D-T image. Therefore, the T-A images are typically used for rapidly detecting possible targets, and the D-T images are adopted in recognizing targets and determining their shapes [1]. The depth-angle (D-A) image (Figure 1C), which defines the abscissa and the ordinate as a beam serial number (corresponding to beam angle) and depth, is also adopt to reduce the size of the D-T image and improve the efficiency of the threshold segmentation. In Figure 1, minimum slant range (MSR) is defined as the minimum radial distance between MBES transducer and seafloor. Because of the interaction of side lobe and seabed echoes, the water column data beyond MSR are seriously polluted. Therefore, in this paper, the detection of gas plume is performed within the MSR.

### 2.2. Characteristics of Noise in the WC Image

**Figure 2:**
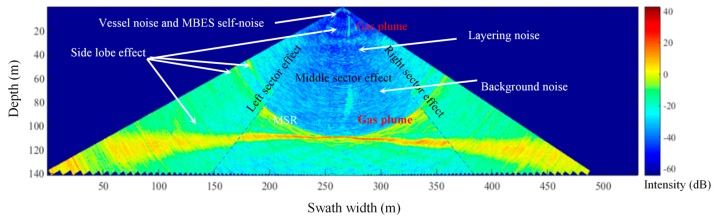
Distributions and characteristics of acoustic components in a WC D-T image.

A MBES transducer recorded echoes including gas plumes, noise caused by the WC environment, interferences from beam side lobes, third-party sonars, and vessels, as well as other targets [1]. Therefore, the interpretation of the image in term of gas plumes is prone to errors. The distributions of noise in the WC image are depicted in Figure 2. The distributions are as follows:

1. Sea surface noise caused by vessel and MBES transducer affects the BSs of the top of a ping.
2. Layering noise is a phenomenon in the WC image and is formed by the echoes from the non-gas plume targets, such as marine organisms, inanimate matter, and the inhomogeneous sea structure [37]. Generally, the noise distributes horizontally in different water layers and exhibits a strong BS in deep layers [26].
3. A side lobe effect is produced by the interference of the side lobes and target echoes. The beam pattern of a conventional Mills Cross MBES is defined by a main lobe and side lobes. The scattering signal of targets can be extended given the presence of the side lobes, and seabed echoes contaminate the WC data at any slant range beyond the closest distance of approach to the seabed [1]. The side lobe effect near a seabed is called the minimum slant range (MSR) effect. The MSR effect implies that the gas plume can be detected efficiently only within the MSR.
4. Sector configuration leads to the difference of noise distributions in a WC image. Because of low emission frequency and long measurement distance, noise in the outside sectors is higher than that in the middle ones.
5. Background noise, which is formed by the echoes from turbulence, particles scattering, non-buoyant microbubbles and so on in water mass [38,39], has the characteristic of discrete distribution in the WC image.

These effects appear with the gas plumes in the WC images and challenge the traditional threshold segmentation methods, such as the 2D Otsu and iterative threshold segmentation methods mentioned in Section 1 in the gas plume detection. Furthermore, the distribution differences of noise in the different types of WC images demonstrated in Figure 1 imply the necessity of removing noise by combining the T-A, D-A, and D-T images.

## 3. Detection of Gas Plumes

Gas plume detection consists of separating the gas plume from the noise in a WC image. Figure 2 illustrates that noise mostly distribute along the depth and horizontal directions and have the BS differences in various sectors in a WC image. Therefore, a detection method based on the characteristics of gas plume and noise is presented in this section. For a sector, three main steps are involved in the method:

Step 1: Threshold segmentation for the T-A and D-A images

The average beam intensity curves of the T-A and D-A images are first calculated, and then the threshold segmentations are conducted for the two images through the mean standard deviation threshold segmentation method to weaken the noise.

Step 2: Intersection calculation of the two segmented images

The segmented D-A image is converted into the T-A image. Then an intersection operation is performed for the two segmented T-A images to maintain the common targets, remove the non-common residual noise, and obtain an intersection image.

Step 3: Morphological constraint of the gas plume

The T-A intersection image is converted into the D-T image to obtain nearly real shapes and positions of targets in the WC image, and then the morphological characteristics of the gas plumes are used as the thresholds to detect the gas plumes in the WC images.

**Figure 3:**
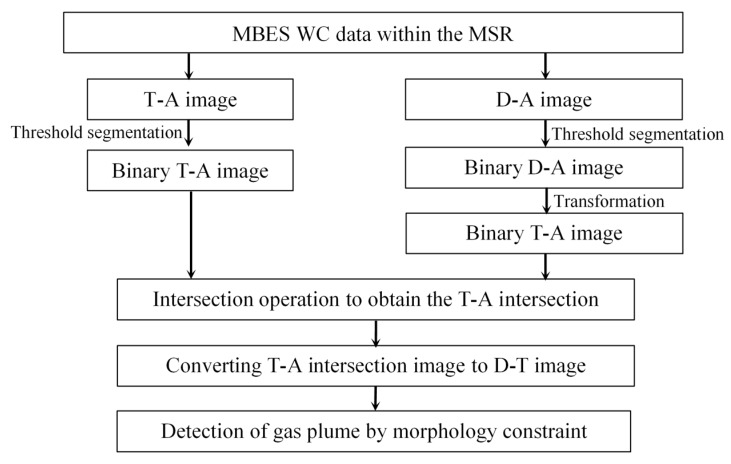
Detection of gas plumes by the proposed method.

The above process is depicted in Figure 3. The same process is used for the other sectors, and the target detection in a ping WC image can be performed.

### 3.1. Mean Standard Deviation Threshold Segmentation Algorithm

Threshold segmentation has a central position in image segmentations given the simplicity and efficiency of this process [40], in which the key is selecting a threshold. The mean standard deviation threshold segmentation algorithm is adopted in this research considering the characteristics of the noise and gas plumes in the WC images. The threshold, *μ* + *kσ*, is determined in a combined manner by the mean *μ* and standard deviation *σ* of the BSs of a sector. The *k* is a constant within 1–3. The determination of *k* is discussed in Section 5.1. The identification of the interesting targets from the noise and interferences by using *μ* + *kσ* follows the principle below:

(1) $$g(x,y)=\{\begin{array}{cc} 1, & f(x,y)>\mu +k\sigma \\ 0, & f(x,y)\leq \mu +k\sigma \end{array}$$

where *f*(*x*, *y*) is the WC data or image, and *g*(*x*, *y*) is the segmented binary image.

### 3.2. Threshold Segmentations for T-A and D-A Images

#### 3.2.1. Threshold Segmentation for T-A Images

**Figure 4:**
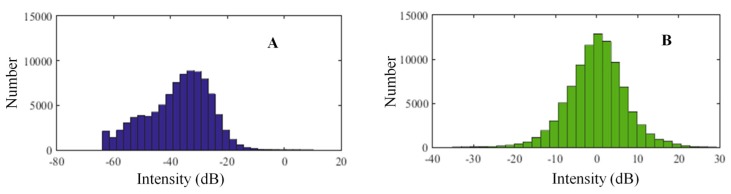
(**A**) Histograms of ***M*** (*a* × *b*) and (**B**) ***M*_1_**(*a* × *b*).

The threshold segmentation must be handled independently in different sectors given the differences in transmission frequencies differences. By using a sector as an example, if the matrix of the BSs of sampling points is ***M*** (*a* × *b*), then the segmentation process is depicted as follows:

1. The mean is calculated by averaging the BSs of each row and a column vector with *a* rows is obtained.
2. The mean BS is subtracted from the raw BSs of each row, and a new matrix ***M*_1_** (*a* × *b*) is acquired. Figure 4 implies that the raw BSs do not obey the normal distribution, whereas ***M*_1_** does. Therefore, the process is called normalization of the T-A image.
3. The threshold *μ* + *kσ* is obtained by calculating the mean and the standard deviation *σ* of ***M*_1_**.
4. ***M*_1_** is smoothened to highlight the targets in the T-A image, and ***M*_2_** (*a* × *b*) is obtained. In the smoothing process, the convolution smoothing is adopted for each column of ***M*_1._** If a column vector is *d*, and the weighted window is *w*, then we obtain the following equation:
  (2) $$(d*w)[n]=\sum_{m=0}^{M-1}d\left[n-m\right]w\left[m\right]\;0\leq n\leq N+M-2,0\leq m\leq M-1$$
  where *d* is the column vector of ***M*_1_**, *N* is the length of *d*, and *w* is the weighted window function with window size *M*. The head and end of the sequence after the convolution should be removed to ensure *N* invariability before and after convolution. A Hanning function is used to eliminate the high-frequency interference from a non-periodic continuous signal and is thus adopted as the weighted window *w*:
  (3) w(m)=0.5−0.5cos(2πm/(M−1))  0≤m≤M−1
5. Target or noise is diagnosed by comparing the value of each sampling point in ***M*_2_** with *μ* + *kσ*. If the value is more than *μ* + *kσ*, then the sampling point is determined as a target point and retained. Otherwise, the sampling point is determined as noise and removed.

All sectors are processed by using these five steps until the segmentation is completed.

**Figure 5:**
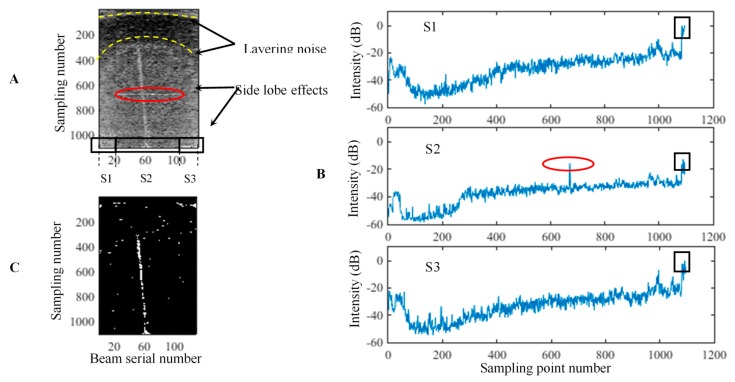
Threshold segmentation for the T-A image. **A**–**C** denote the raw T-A image within the MSR, mean BS curves of the three sectors, and segmented T-A image. **S1**–**S3** are the three sectors in the T-A image. The circles and rectangles indicate the side lobe effects in different water depths, whereas the arcs represent the layering noise.

Figure 5 depicts the process. The figure illustrates the T-A image formed by the raw WC data within the MSR. Moreover, the high noise appear in the two outside sectors, the arc-shaped layering noise distributes in the different water layers, and the side lobe effects accompany the gas plume and distribute horizontally in the different water layers. In Figure 5B, the mean BS curves of the three sectors reflect the noise. The layering noise is reflected in the BS variations of the different sampling numbers, whereas the side lobe effects are shown in the black rectangles and red circle. Figure 5C indicates that the side lobe effects and background noise are mostly removed after the threshold segmentation, whereas the layering noise is weakened.

#### 3.2.2. Threshold Segmentation for D-A Image

The D-A image differs from the corresponding T-A image in the displayed form of a ping fan. In the D-A image, the layering noise and side lobe effects are presented as horizontal and arc-shaped distributions in different depths, respectively. Therefore, the layering noise that remains partly in the segmented T-A image can be removed efficiently from the D-A image by using the threshold segmentation. If the raw BS matrix of a sector in the D-A image is ***N*** (*a*_1_ × *b*_1_), then the threshold segmentation follows similar steps as described in Section 3.2.1. All sectors are segmented by the five steps, and the segmentation is performed in the D-A image.

**Figure 6:**
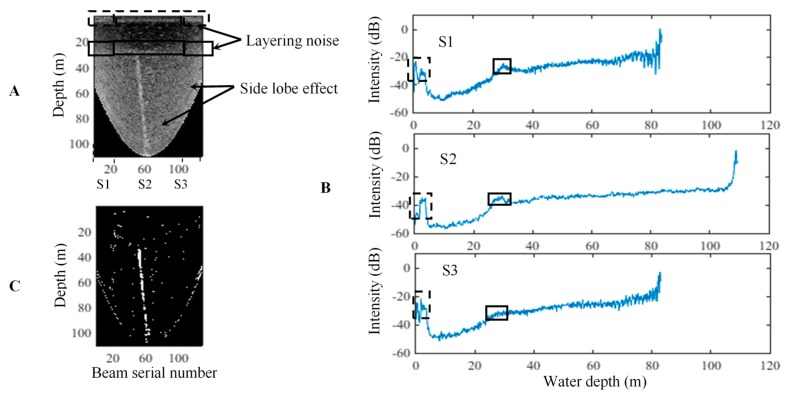
Threshold segmentation for the D-A image. (**A**–**C**) denote the raw D-A image within the MSR, mean BS curves of the three sectors, and segmented D-A image. **S1**–**S3** are the three sectors in the ping fan. The rectangles indicate the layering noise of the different water depths reflected in the mean BS curves.

Figure 6 demonstrates the threshold segmentation process of the D-A image. The raw ping WC data used in Figure 5 is extracted to form the D-A image (Figure 6A). The D-A image also indicates that high noise is found in the two outside sectors. The layering noise distributes horizontally, and the side lobe effects display as the arc-shaped distributions. The distributions of the two types of noise are reversed compared with the distributions depicted in Figure 5A. The noise can also be effectively reflected in the mean BS curves (Figure 6B). The layering noise and the background noise are mostly removed after the threshold segmentation (Figure 6C), whereas the side lobe effects are weakened.

### 3.3. Intersection Operation for the Two Segmented Images

The background noise can be mostly removed via the above threshold segmentation for the T-A and D-A images. The segmentation for the T-A image mainly removes the side lobe effects but remains the residual layering noise in the segmented T-A image, whereas the segmentation for the D-A image eliminates the layering noise but keeps the residual side lobe effects in the segmented D-A image. The two segmentations indicate complementarity. Therefore, an intersection operation for the segmented T-A and D-A images may be performed to eliminate most residual noise from the two segmented images. Because the segmented binary D-A image differs from the segmented binary T-A image, the segmented binary D-A image need to be converted to a new binary T-A image for the intersection operation. The intersection operation of the segmented and the new T-A images can be expressed as:

(4) $$G(x,y)=g_{1}(x,y)\cap g_{2}(x,y)=\{\begin{array}{cc} 1, & g_{1}(x_{i},y_{j})=1\;\&\;g_{2}(x_{i},y_{j})=1\\ 0, & \text{other} \end{array}$$

where *G* (*x*, *y*) is the intersection image of the two T-A images, *g*^1^ (*x*, *y*) is the segmented T-A image, and *g*^2^ (*x*, *y*) is the new T-A image. 0 means noise and 1 means target. The two images exhibit the same size *m* × *n*, (*i*, ⊂*j*)(*m*, *n*).

In the intersection operation, if a target appears at the same position of the two segmented T-A images, then this target will remain in the final intersection image; otherwise, it will be removed as a noise. Therefore, the intersection operation retains the common targets and removes the residual noise in the two images.

### 3.4. Gas Plume Detection Based on Morphology Characteristics 

At this point, most of the noise in the WC data are eliminated, and the targets remain in a ping image after the abovementioned process. However, some noise whose BSs are high remain in the intersection image because the mean standard deviation threshold segmentation method is based on BS feature of gas plumes. Therefore, we need to use other characteristics such as positional and morphological features of gas plumes to detect and recognize gas plumes from the D-T image of intersection image:

(1) Position features

Gas plumes that rise from the seafloor usually dissolve during the rising process, and cannot reach the sea surface. We can set a depth threshold to reduce the detection range. Setting the threshold needs to consider depth variations of gas plumes in a detected water area and completely remove the effect of residual water surface noise. Generally, the threshold is set as more than 5 m underneath the MBES transducer.

(2) Morphological feature

The flare-shape of gas plumes is different from noise [41]. We can use morphological features such as height, area, and width to distinguish gas plumes from noise. The characteristics of gas plumes mentioned in Section 1 show that the heights of gas plumes are various, and higher than those of noise. The flare-shape gas plumes often present nearly linear distribution. We can define a linear structuring element ***B*** = strel(‘line’, len, deg), then conduct first closing and second opening operations [40] to image ***A***, namely the D-T image of intersection image in MATLAB:

(5) $$\begin{array}{l} A\circ B=(A⊙B)⊕B\\ A•B=(A⊕B)⊙B\\ (A•B)\circ B=\{[\left(A⊕B\right)⊙B]⊙B\}⊕B \end{array}$$

where ∘ and • are opening and closing operations and ⊕ and ⊙ denote the dilation and erosion operations, respectively.

The strel class in MATLAB is used to define morphological structuring element, strel (‘line’ len, deg) creates a linear structuring element, deg specifies the angle (in degrees) of the line, and len is approximately the distance between the centers of the structuring element members at opposite ends of the line. The deg is set to 90, and len is set by the minimum detection height that refers the detection requirement and resolution of the WC data in the study.

The area of a flare-shaped gas plume is generally greater than that of noise. Area threshold, namely the minimum detection area (height × width), are set by referring to the detection requirement and resolution of the WC data. The minimum detection height and width can be calculated quantitatively following the description in Section 5.4.

## 4. Experiments and Analysis

### 4.1. Experiment I: Detections of Gas Plumes from Shallow Water EM710 WC Data

A shallow-water MBES measurement was performed in the water area with 0.5 km × 1.5 km, roughly 160 m of water depth, and dense gas plumes in June 2012, in the South China Sea to verify the proposed method. In the MBES measurement, a Kongsberg EM 710 device was adopted. A total of 120° of the swath coverage, three sectors, 128 beams in a ping fan, 6944.44 Hz of sampling frequency were used in a beam measurement. The dual-swath mode was set in EM 710 WC data acquisition. The dual-swath mode sets different transmission frequencies for different sectors and adjacent pings. For an odd ping, the frequencies of the middle and two outside sectors are 81 and 73 kHz, correspondingly, whereas are 97 and 89 kHz, respectively, for the even ping. A 1.6 km-length surveying line with roughly 500 m swath width, which covers the water area, was designed and 1444 pings of WC data were recorded by the Kongsberg Seafloor Information System (SIS) data acquisition software developed by Kongsberg Maritime Company at about 2.2 m of ping interval. By decoding the binary WC data files with the suffix *.wcd and performing data processing by the process mentioned in Section 2.1, WC images of recorded pings are formed by self-developed software. Based on these parameters of EM 710, the size of the detected minimum target is defined as 0.2 m in height, 2.6 m in width, and 2 m^2^ in area, correspondingly by referring to Section 5.4 for later detections of gas plumes.

**Figure 7:**
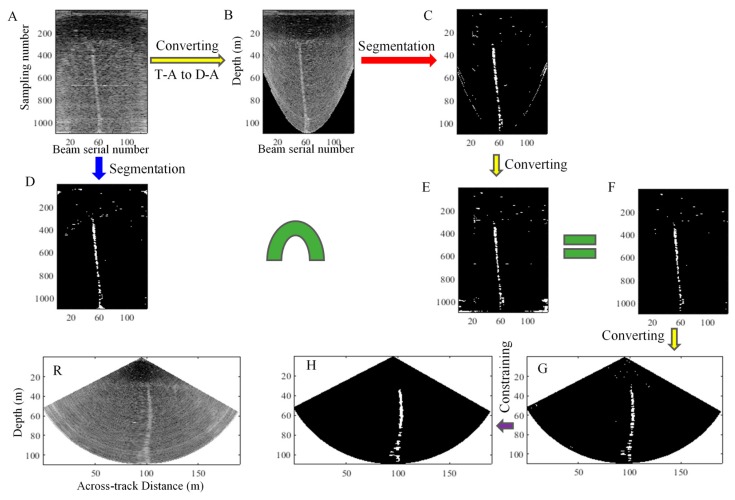
Process of gas plume detection in a ping fan through the proposed method. (**R**) Raw D-T image of the 112th ping fan; (**A**) T-A image; (**D**) Segmented T-A image; (**B**) D-A image formed by converting the T-A image; (**C**) Segmented D-A image; (**E**) New T-A image formed by an image transformation from (**C**); (**F**) Intersection image obtained by performing an intersection operation to (**D**,**E**); (**G**) D-T image obtained by an image transformation from (F); (**H**) Gas plumes detected by the morphological constraint to (**G**). A, D, E, and F are the T-A images and have the same axes and labels. B and C are the D-A images and have the same axes and labels. R, G, and H are the D-T images and have the same axes and labels.

The 112th ping with a gas plume is extracted for the detection via the proposed method, and the *k* in the threshold *μ* + *kσ* is set as 1.5. The process is depicted in Figure 7. The results are presented in Figure 7F. In this figure, slight amounts of noise remain. This phenomenon is caused by the threshold segmentation algorithm. Most noise is removed because of its BS that is less than the given threshold, whereas the retained noise demonstrate higher BSs than the threshold. The morphological constraint (detailed in Section 3.4) effectively compensates the limitations of the algorithm and detects the gas plumes, as depicted in Figure 7H.

All the 1444 pings in the surveying line are processed by using the same steps as mentioned above. The total correct detection rate *γ* and the gas plumes correct detection rate *γ_gas_plumes_* are calculated to quantitatively assess the performance of the proposed method and defined as [42]:

(6) $$\gamma =(N_{c}/N_{all})\times 100\%$$

(7) $$\gamma_{gas\_plumes}=(N_{gas\_plumes}/N_{manual\_gas\_plumes})\times 100\%$$

where *N_all_* is the ping number of the entire survey line, *N_manual_gas_plumes_* is the number of pings that contain gas plumes and is obtained by manual inspection, *N_gas_plumes_* is the number of pings with gas plumes and is detected by the proposed method, and *N_c_* is the correct detection number of pings, including gas plumes and null pings by the proposed method. In the experiment, the proposed method fulfills the automatic detection and achieves *γ* ≈ 86% and *γ_gas_plumes_* ≈ 86%, thereby indicating that the proposed method is efficient in detecting gas plumes from the seriously polluted shallow-water WC images.

**Figure 8:**
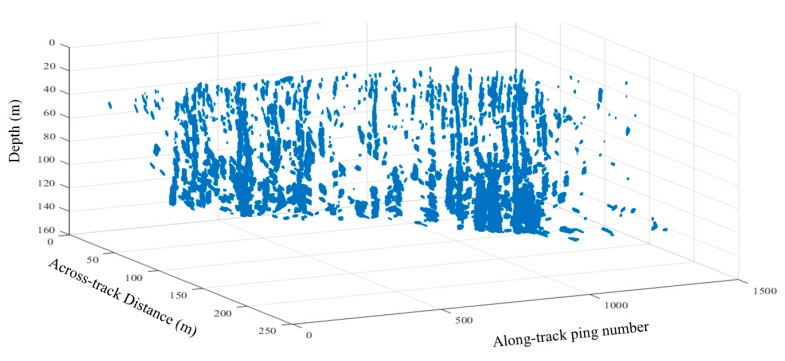
Distributions and shapes of the gas plumes detected in shallow water.

Based on the detection, the distributions of the gas plumes in the surveying area are illustrated in Figure 8. The 3D shape of each gas plume is also drawn by the 3D coordinates of all the echoes from the detected gas plumes. The gas plumes distribute densely in the area and have heights that vary from several meters to approximately 50 m.

### 4.2. Experiment II: Detection of Gas Plume from Deepwater EM122 WC Data

A deep-water MBES measurement was performed in the water area with 3.0 km × 40 km, roughly 1500 m of water depth and sparse gas plumes in March 2016, in the South China Sea to further verify the proposed method. In the measurement, a Kongsberg EM 122 unit was adopted. The swath coverage of 90°, six sectors, 288 beams in a ping fan, 67.34 Hz of sampling frequency in a beam measurement, dual-swath mode are set in EM 122 WC data acquisition. In the dual-swath mode, the frequencies of sectors 1–6 are 11, 12.8, 12.2, 12.95, 12.35, and 11.75 kHz correspondingly for an odd ping, and are 11.3, 13.1, 12.5, 13.25, 12.65, and 12.05 kHz for the even ping. A 40 km-length surveying line with roughly 3000 m swath width, covering the water area, was designed and 5960 pings of WC data were recorded by Kongsberg SIS at about 13 m of ping interval. By a similar process as used in the shallow-water MBES WC data, the BSs, gray levels and positions of echoes in a ping are obtained and WC images of recorded pings are formed. The height, width, and area of the detected minimum target are defined as 11.3 m, 10.5 m, and 230 m^2^, respectively, by referring these performance parameters of EM 122 for later detections of gas plumes based on morphological constraints.

**Figure 9:**
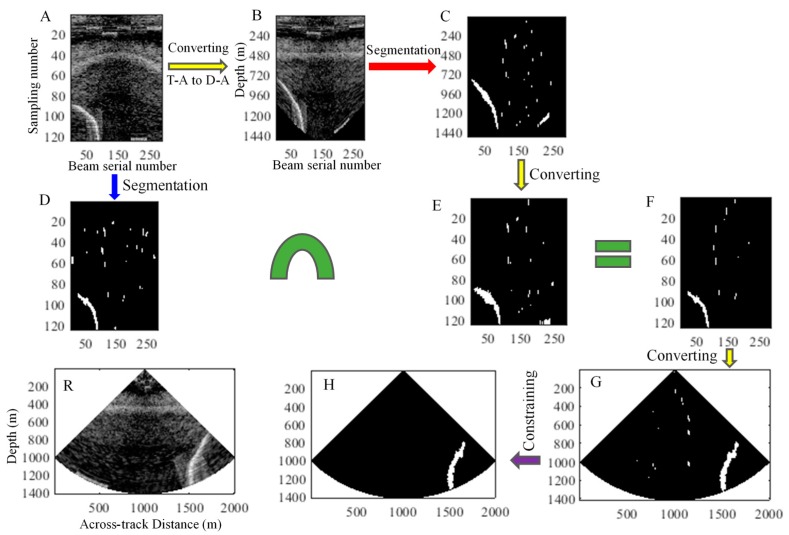
Process of gas plume detection in the 2513th ping through the proposed method. (**A**–**R**) are images that are similar to those presented in (**A**–**R**) of Figure 7.

The 2513th ping with a gas plume is extracted for the detection through the proposed method and the *k* in the threshold *μ* + *kσ* is set as 1.2. A process similar to the one shown in Figure 7 is depicted in Figure 9. The layering phenomenon of noise becomes more significant, and the intensities of noise are higher than those in shallow-water images. The same process is adopted in the detection; the results (Figure 9H) display the detected gas plume clearly and accurately.

**Figure 10:**
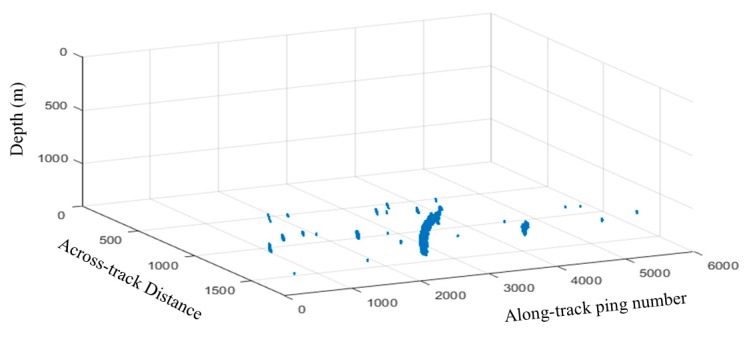
Distributions and shapes of gas plumes detected in deepwater.

We achieved *γ* ≈ 99%, *γ_gas_plumes_* ≈ 80% when detect all 5960 pings (Figure 10). *γ* is higher than *γ_gas_plumes_* because of only 30 pings including gas plumes, and *γ_gas_plumes_* is lower than that in Experiment I because of more complicated noise and interferences. The threshold segment method and detection process are similar to those used in Experiment I. The result shows that the proposed method is efficient in the shallow-water and deepwater detections of gas plumes.

## 5. Discussion

### 5.1. Determination of k

**Figure 11:**
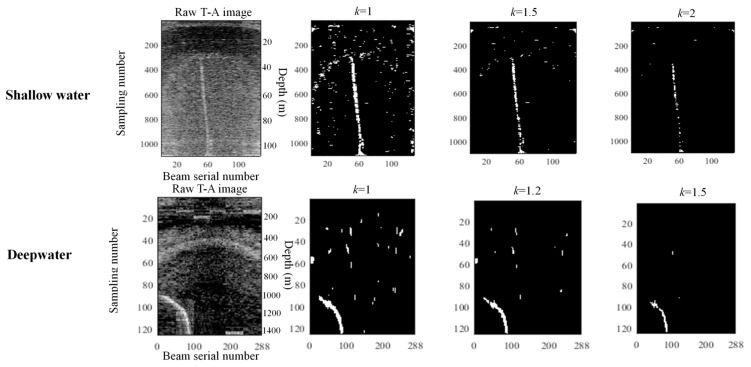
Selection of *k* in the threshold segmentation.

*k* has a significant effect on the threshold segmentation. The confidence coefficient of a detected gas plume is high when the *k* is higher, but missing small gas plumes is possible. Conversely, some noise may be diagnosed mistakenly as gas plumes when *k* is too small. *k* is a constant that varies from 1 to 3. The noise is mainly produced by the marine organism, suspended solids, and so on. The distributions of marine organisms vary with marine temperature profile, demonstrate dense in shallow water and sparse in deep water (Figure 11). The suspended solids, influenced by buoyance, mainly distribute in shallow water. The above results in that the noise in deepwater WC images differ from that in shallow water WC images. This phenomenon has been proven by the above experiments and implies that *k* should vary with water depths. Many segmentation experiments for detecting gas plumes in shallow-water and deepwater WC images are conducted under different *k* to obtain an appropriate *k*. Figure 11 shows the three classic results at *k* as 1, 1.5, and 2 in shallow-water experiments and shows that *k* = 1.5 is the best. The result at *k* as 1 includes considerable noise because of the calculated threshold being too small, whereas the excessively large threshold (*k* as 2) leads to the segmented gas plume scattering. Therefore, *k* is set as 1.5 in the shallow-water detections. Similar experiments are performed for the deepwater WC images, and 1.2 is selected as the optimum *k*.

### 5.2. Threshold Segmentation Methods

**Figure 12:**
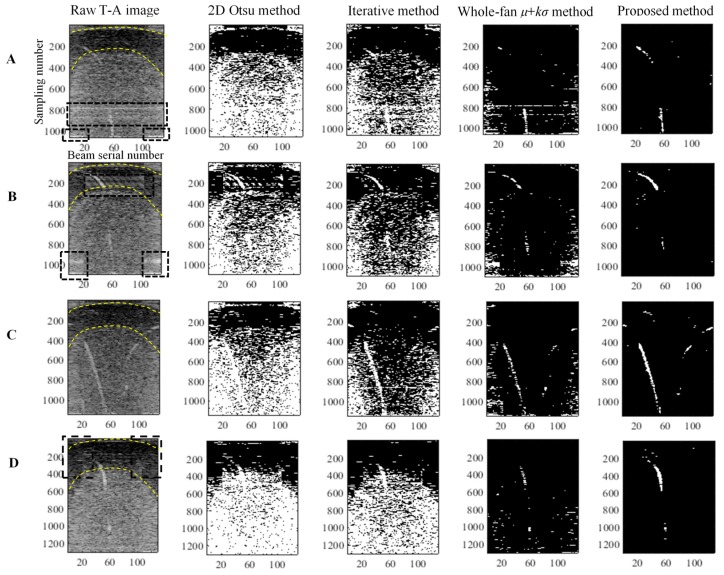
Threshold segmentations for different-ping T-A images through the 2D Otsu, iterative, whole-fan *μ* + *kσ* threshold segmentation, and the proposed methods. (**A**–**D**) denote the four ping fans. The rectangles and arcs represent the side lobe effects and layering noise, respectively. All the images have the same axes and labels as the raw T-A image.

Four representative ping T-A images in Experiment I, A–D, are extracted and segmented via the proposed, whole-fan *μ* + *kσ* threshold segmentation, 2D Otsu, and iterative methods. The results are shown in Figure 12. The four images are seriously polluted by noise. The highlighted linear-shaped noise caused by the side lobe effects and marked with the rectangles appears clearly in A and B but unclearly in C and D. The arc-shaped layering noise marked with arc lines and induced by the inhomogeneous sea structure and distributions of marine organisms are displayed in each ping image. The segmentation results show that the 2D Otsu and iterative methods nearly cannot distinguish the gas plumes from their background. The whole-fan *μ* + *kσ* method has better performance than the two methods, but the results remain unsatisfactory. The proposed method achieved the best segmentations because the 2D Otsu and iterative methods ignore the characteristics of the noise and handle these T-A images as normal optical image. Thus, the background noise is only weakened, whereas considerable noise remains in the segmented images. The discrete arc-shaped layering noises are still in the segmented T-A image, although the whole-fan *μ* + *kσ* segmentation method segments the images along the beam directions and weakens the linear-shaped side lobe effects. The reason is that the method disregards the frequency and noise differences among adjacent sectors, and the method performs the threshold segmentation in the whole fan with an invariable threshold. However, the proposed method nearly eliminates all the noise and clearly presents the gas plumes in the last binary T-A images.

The gas plumes in Experiments I and II are detected by using the four methods and combining the intersection operation and morphological constraint. The results are listed in Table 1. The proposed method performs better than the other methods do.

### 5.3. Necessity of Segmenting T-A and D-A Images

**Figure 13:**
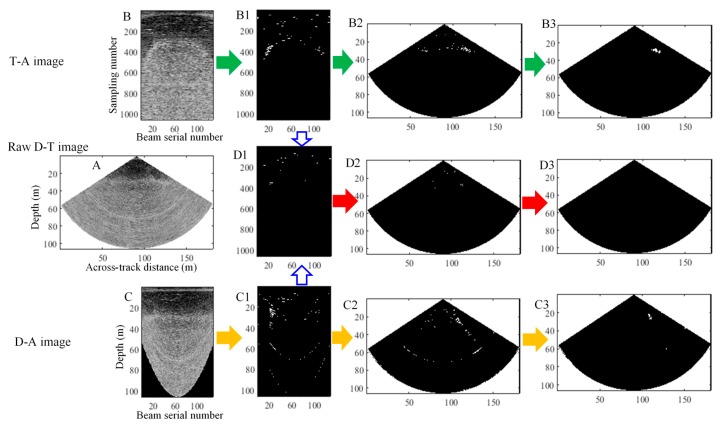
Detections of gas plumes using the T-A image, D-A image, and both. **A** is the raw D-T image; **B** is the raw T-A image; **B1** and **B2** are the segmented T-A and D-T images, respectively; **B3** shows the detection result after the morphological constraint of gas plumes. The corresponding results for the raw D-A image shown in C are displayed in **C1**–**C3**; **D1** is the intersection image of **B1** and **C1**; **D2** is the D-T image of **D1**, and the final detection result from **D2** is shown in **D3**. **B1** and **D1** are the T-A images and have the same axes and labels as **B**. **C** and **C1** are the D-A images and have the same axes and labels. **B2**, **B3**, **D2**, **D3**, **C2**, and **C3** are the D-T images and have the same axes and labels as **A**.

The 39th ping data with obvious noise are extracted for the three experiments below to explain the necessity of segmenting T-A and D-A images. In Experiment 1, we perform the threshold segmentation for the raw T-A image (Figure 13B) and obtain the segmented T-A image (Figure 13B1), transform it into the D-T image (Figure 13B2), and detect a target by using the morphological constraint (Figure 13B3). In Experiment 2, the same process is conducted for the raw D-A image (Figure 13C), and the corresponding results are shown in C1–C3. In Experiment 3, we perform an intersection operation for Figure 13(B1,C1) and obtain the intersection image (Figure 13(D1)). Then, we adopt the same transformation and constraint as those in Experiment 1 and do not detect any target.

In the raw D-T image (Figure 13A), we cannot find any targets, such as gas plumes. The side lobe effects with horizontal distribution and layering noise with arc-shaped distribution can be seen in the T-A image, respectively; the side lobe effects with arc-shaped distribution and layering noise with horizontal distribution can also be observed in the D-A image. Based on different description forms of side lobe effects and layering noise in T-A and D-A images, the threshold segmentation for T-A image completely removes the side lobe effect and attenuates the layering noise, whereas the layering noise is eliminated completely, and the side lobe effects are weakened in the D-A image segmentation. The segmentation results are shown in Figure 13(B1,C1). The detection results (Figure 13(B3,C3)) from the T-A or D-A images are incorrect, thereby indicating that a single T-A or D-A image is insufficient in detecting gas plumes. In the intersection image, the side lobe effect and layering noise are eliminated completely. Only certain scattering noise remains in the image. We do not detect any target in Figure 13(D3) after the morphological constraint. The result is consistent with the manual detection from the raw D-T image and further shows that the T-A and D-A images must be combined to detect the gas plume from a ping fan.

### 5.4. Limitations of the Proposed Method

The proposed method achieves enhanced detection results in the above experiments but may be affected by the following factors:

(1) Shape and size of the gas plume
  The detection capability of the proposed method depends on the MBES performance parameters, water depth, and beam angle. In a beam ray, a small target can be detected by at least two echoes. We use three echoes to detect the small target and ensure the reliability of the detection. The detection resolution, namely, the height and width of a small target can be determined by:
  (8) $$h=l\cos\theta_{i}$$
  (9) $$w=L_{i}\times (\theta_{i}-\theta_{i+1})$$
  where *l* is the distance of the three echoes in the beam ray, *L_i_* is the length of *i*th beam ray, and *θ_i_* and *θ_i_*_+1_ are the *i*th and *I* + 1th beam angles, correspondingly. *L_i_* can be calculated by the water depth *D* divided by cos*θ_i_*. Therefore, the height, width, and area of the detected minimum target are set as 0.2 m, 2.6 m, and 2 m^2^, respectively, in Experiment I and 11.3 m, 10.5 m, and 230 m^2^, correspondingly, in Experiment II. The proposed method is inefficient for the detections of gas plumes less than the minimum target. The problem can be solved by the increase of sampling rate.
(2) MBES WC data
  The proposed method is verified by EM 710 and EM122 WC data and needs to be tested further by other MBES WC data with different performances. In the experiments of this research, the WC data measured by the multi-sector, and dual-swath MBES are adopted. The proposed method is also suitable for detecting targets from the same MBES WC and single-frequency and single-sector WC data.
(3) Similar targets as gas plumes
  The similar targets as gas plumes, such as fishes or fish schools and suspended solids may affect detection of gas plumes. Literature [41,43,44] provided fish-gas discriminate protocols, quantification of seep-related methane gas emissions, and a means to quantify and assess fishes or fish schools in the presence of gas bubbles which may be useful for distinguish gas plume from fishes. In this paper, the BS and morphological features of different targets are used as thresholds to distinguish gas plumes from others. In the extreme case that the threshold parameters of gas plumes and similar targets are close, the diagnosis may be fulfilled by further mining the characteristics of gas plumes and similar targets.
(4) Targets outside of the MSR
  In the proposed method, the WC data outside of the MSR is eliminated because of the serious MSR effect, and only the WC data in the MSR are used for the detection. The WC data in the MSR are also adopted in the studies depicted in the literature [1,26,27]. Existing studies and the proposed method may become ineffective in detecting the targets outside of the MSR.

## 6. Conclusions and Suggestions

The detection method proposed in this paper detects gas plumes from the polluted WC images via three main steps including the mean standard deviation threshold segmentation algorithm for two small-size T-A and D-A images, intersection operation to the two segmented T-A images, and morphological constraint to the intersection image. The proposed method fulfills the automatic detection and achieves higher correct detection rate than the traditional threshold segmentation methods, even for the WC data seriously polluted.

In the applications of the proposed method, some key points should be noted. (1) The parameter *k* has a significant effect on the threshold segmentation and needs to be determined by experiments in the detected water area; (2) Our method is also appropriate for the MBES containing only one sector; (3) Only the gas plume within the MSR is considered for now, and the WC data outside the MSR is ignored; (4) The morphologic constraints (area, height, width) need to be chosen carefully, which can be calculated by Section 5.4(1) or properly specified.

## Figures and Tables

**Table 1:** Comparisons of various methods. The results before and after “/” are obtained by using the WC data of Experiments I and II. The parameters are achieved under the same calculating conditions.

Parameter	2D Otsu	Iteration	Whole-Fan *μ* + *kσ*	Proposed Method
Correct detection rate (%)	22/36	14/28	9/60	86/99
Correct detection rate of gas plumes (%)	3/10	1/10	10/4	86/80
Time consumed (min)	112/173	116/196	101/181	109/177

## References

[1] Hughes Clarke J.E. (2006). Applications of multibeam water column imaging for hydrographic survey. Hydrogr. J..

[2] Nikolovska A., Sahling H., Bohrmann G. (2008). Hydroacoustic methodology for detection, localization, and quantification of gas bubbles rising from the seafloor at gas seeps from the eastern black sea. Geochem. Geophys. Geosyst..

[3] Weber T., Mayer L.A., Beaudoin J., Jerram K., Malik M.A., Shedd B., Rice G. (2012). Mapping gas seeps with the deepwater multibeam echosounder on okeanos explorer. Oceanography.

[4] Weber T.C., Peña H., Jech J.M. (2009). Consecutive acoustic observations of an atlantic herring school in the northwest atlantic. ICES J. Mar. Sci. J. Cons..

[5] Trevorrow M.V. (2005). Observations of acoustic scattering from turbulent microstructure in knight inlet. Acoust. Res. Lett. Online.

[6] Buelens B., Pauly T., Williams R., Sale A. (2009). Kernel methods for the detection and classification of fish schools in single-beam and multibeam acoustic data. ICES J. Mar. Sci. J. Cons..

[7] Melvin G.D., Cochrane N.A. (2015). Multibeam acoustic detection of fish and water column targets at high-flow sites. Estuar. Coasts.

[8] Innangi S., Bonanno A., Tonielli R., Gerlotto F., Innangi M., Mazzola S. (2016). High resolution 3-d shapes of fish schools: A new method to use the water column backscatter from hydrographic multibeam echo sounders. Appl. Acoust..

[9] Hughes Clarke J.E., Lamplugh M., Czotter K. Multibeam Water Column Imaging: Improved Wreck Least-Depth Determination. Proceedings of the Canadian Hydrographic Conference.

[10] Van der Werf A. (2010). Mast Tracking Capability of em 3002 d Using Water Column Imaging.

[11] Wyllie K., Weber T., Armstrong A. Using multibeam echosounders for hydrographic surveying in the water column: Estimating wreck least depths. Proceedings of the US Hydrographic Conference.

[12] Norton A.R., Dijkstra S. Developments in eelgrass mapping methodology using hydrographic multi-beam sonar. Proceedings of the 25th Annual Zosterapalooza.

[13] Schneider von Deimling J., Weinrebe W. (2014). Beyond bathymetry: Water column imaging with multibeam echo sounder systems. Hydrogr. Nachr..

[14] Gardner J.V., Malik M., Walker S. (2009). Plume 1400 meters high discovered at the seafloor off the northern california margin. Eos Trans. Am. Geophys. Union.

[15] Yang F.L., Han L.T., Wang R.F., Shi B. (2013). Progress in object detection in middle and bottom-water based on multibeam water column image. J. Shandong Univ. Sci. Technol. Nat. Sci..

[16] Colbo K., Ross T., Brown C., Weber T. (2014). A review of oceanographic applications of water column data from multibeam echosounders. Estuar. Coast. Shelf Sci..

[17] Lee S.Y., Holder G.D. (2001). Methane hydrates potential as a future energy source. Fuel Process. Technol..

[18] Schneider von Deimling J., Brockhoff J., Greinert J. (2007). Flare imaging with multibeam systems: Data processing for bubble detection at seeps. Geochem. Geophys. Geosyst..

[19] Jordt A., Zelenka C., von Deimling J.S., Koch R., Köser K. (2015). The bubble box: Towards an automated visual sensor for 3d analysis and characterization of marine gas release sites. Sensors.

[20] Boelmann J., Zielinski O. (2015). Automated characterization and quantification of hydrocarbon seeps based on frontal illuminated video observations. J. Eur. Opt. Soc. Rapid Publ..

[21] Klaucke I., Weinrebe W., Petersen C.J., Bowden D. (2010). Temporal variability of gas seeps offshore new zealand: Multi-frequency geoacoustic imaging of the wairarapa area, hikurangi margin. Mar. Geol..

[22] Leifer I., Patro R.K. (2002). The bubble mechanism for methane transport from the shallow sea bed to the surface: A review and sensitivity study. Cont. Shelf Res..

[23] Weber T.C., Jerram K., Mayer L. (2012). Acoustic sensing of gas seeps in the deep ocean with split-beam echosounders. Proc. Meet. Acoust..

[24] Sahling H., Römer M., Pape T., Bergès B., Dos S.F.C., Boelmann J., Geprägs P., Tomczyk M., Nowald N., Dimmler W. (2014). Gas emissions at the continental margin west off svalbard: Mapping, sampling, and quantification. Biogeosci. Discuss..

[25] Veloso M., Greinert J., Mienert J., De Batist M. (2015). A new methodology for quantifying bubble flow rates in deep water using splitbeam echosounders: Examples from the arctic offshore nw-svalbard. Limnol. Oceanogr. Methods.

[26] Marques C.R., Hughes Clarke J.E. Automatic mid-water target tracking using multibeam water column. Proceedings of the Canadian Hydrographic Conference: The Arctic, Old Challenges, New Approaches.

[27] Urban P., Köser K., Greinert J. (2017). Processing of multibeam water column image data for automated bubble/seep detection and repeated mapping. Limnol. Oceanogr. Methods.

[28] Gonzalez R.C., Woods R.E. (2011). Digital Image Processing.

[29] Goswami R., Rao G.S.B., Rani S.S., Kumar M., Prasad S.D., Kumar G.H. (2010). Segmentation of sonar images based on adaptive thresholding with image histogram. Digit. Image Process..

[30] Collier J.S., Humber S.R. (2007). Time-lapse side-scan sonar imaging of bleached coral reefs: A case study from the seychelles. Remote Sens. Environ..

[31] Degraer S., Moerkerke G., Rabaut M., Van Hoey G., Du Four I., Vincx M., Henriet J.-P., Van Lancker V. (2008). Very-high resolution side-scan sonar mapping of biogenic reefs of the tube-worm lanice conchilega. Remote Sens. Environ..

[32] Zhao J., Yan J., Zhang H., Meng J. (2017). A new radiometric correction method for side-scan sonar images in consideration of seabed sediment variation. Remote Sens..

[33] Zhao J., Wang X., Zhang H., Hu J., Jian X. (2016). Side scan sonar image segmentation based on neutrosophic set and quantum-behaved particle swarm optimization algorithm. Mar. Geophys. Res..

[34] Brook A., Ben-Dor E. (2011). Automatic registration of airborne and spaceborne images by topology map matching with surf processor algorithm. Remote Sens..

[35] Fakiris E., Papatheodorou G., Geraga M., Ferentinos G. (2016). An automatic target detection algorithm for swath sonar backscatter imagery, using image texture and independent component analysis. Remote Sens..

[36] Kongsberg Kongsberg em Series Multibeam Echo Sounder em Datagram Formats. https://www.km.kongsberg.com/ks/web/nokbg0397.nsf/AllWeb/253E4C58DB98DDA4C1256D790048373B/$file/160692_em_datagram_formats.pdf.

[37] Waite A. (2002). Sonar for Practising Engineers.

[38] Ross T., Lueck R. (2003). Sound scattering from oceanic turbulence. Geophys. Res. Lett..

[39] Best J.L., Parsons D.R., Simmons S.M., Oberg K.A., Johnson K.K., Czuba J.A., Malzone C. (2008). Coherent flow structures over alluvial sand dunes revealed by multibeam echo sounding. Mar. River Dune Dyn..

[40] Gonzalez R.C., Woods R.E., Eddins S.L. (2013). Digital Image Processing Using Matlab.

[41] Judd A., Davies G., Wilson J., Holmes R., Baron G., Bryden I. (1997). Contributions to atmospheric methane by natural seepages on the U.K. Continental shelf. Mar. Geol..

[42] Powers D.M. Evaluation: From Precision, Recall and f-Measure to Roc, Informedness, Markedness and Correlation. http://hdl.handle.net/2328/27165.

[43] Ostrovsky I. (2009). Hydroacoustic assessment of fish abundance in the presence of gas bubbles. Limnol. Oceanogr. Methods.

[44] Von Deimling J.S., Rehder G., Greinert J., McGinnnis D., Boetius A., Linke P. (2011). Quantification of seep-related methane gas emissions at tommeliten, north sea. Cont. Shelf Res..

